# Immune interconnectivity of anatomically distant tumors as a potential mediator of systemic responses to local therapy

**DOI:** 10.1038/s41598-018-27718-1

**Published:** 2018-06-21

**Authors:** Rachel Walker, Jan Poleszczuk, Shari Pilon-Thomas, Sungjune Kim, Alexander A. R. A. Anderson, Brian J. Czerniecki, Louis B. Harrison, Eduardo G. Moros, Heiko Enderling

**Affiliations:** 1 Department of Integrated Mathematical Oncology, H. Lee Moffitt Cancer Center and Research Institute, Tampa, FL USA; 2 Nalecz Institute of Biocybernetics and Biomedical Engineering, Polish Academy of Sciences, Warsaw, Poland; 3 Department of Immunology, H. Lee Moffitt Cancer Center and Research Institute, Tampa, FL USA; 4 Department of Radiation Oncology, H. Lee Moffitt Cancer Center and Research Institute, Tampa, FL USA; 5 Department of Breast Oncology, H. Lee Moffitt Cancer Center and Research Institute, Tampa, FL USA

## Abstract

Complex interactions occur between tumor and host immune system at each site in the metastatic setting, the outcome of which can determine behavior ranging from dormancy to rapid growth. An additional layer of complexity arises from the understanding that cytotoxic T cells can traffic through the host circulatory system. Coupling mathematical models of local tumor-immune dynamics and systemic T cell trafficking allows us to simulate the evolution of tumor and immune cell populations in anatomically distant sites following local therapy and thus computationally evaluate immune interconnectivity. Results suggest that the presence of a secondary site may either inhibit or promote growth of the primary, depending on the capacity for immune recruitment of each tumor and the resulting systemic redistribution of T cells. Treatment such as surgical resection and radiotherapy can be simulated to estimate both the decrease in tumor volume at the local treatment-targeted site, and the change in overall tumor burden and tumor growth trajectories across all sites. Qualitatively similar responses of distant tumors to local therapy (positive and negative abscopal effects) to those reported in the clinical setting were observed. Such findings may facilitate an improved understanding of general disease kinetics in the metastatic setting: if metastatic sites are interconnected through the immune system, truly local therapy does not exist.

## Introduction

Tumor-immune interactions (or cancer immunoediting) can lead to either cancer elimination by the immune system, tumor dormancy achieved by a complex equilibrium between tumor and immune cells, or tumor escape^1,2^. Tumor-associated antigens, stress proteins, and danger-associated molecular patterns are endogenous immune adjuvants that can both initiate and continually stimulate an immune response against a tumor^3,4^. In retaliation, tumors can hijack intrinsic immune regulatory programs that are intended to prevent autoimmune disease^5^, thereby facilitating continued growth despite an activated antitumor immune response. In metastatic disease, this ongoing tumor-immune battle occurs at each site. Adding an additional layer of complexity, there has been mounting evidence in recent years that these interactions also occur systemically, with locally activated cytotoxic T cells trafficking through the host circulatory system and surveilling metastases elsewhere in the body^6^. Thus, metastatic tumors may be highly interdependent; changes in the tumor-immune interactions at one site could perturb the antitumor immune response systemically.

The abscopal effect – local treatment-induced responses in distant, untreated metastatic sites – is becoming more widely accepted following several prominent clinical studies and case reports. In a retrospective analysis of pre-and post-therapy radiology images of 47 metastatic melanoma cases treated with both ipilimumab and 65 courses of radiation, a shrinking of tumor lesions outside the radiation field was observed in 16 patients, the majority of which had exhibited continued growth after immunotherapy alone^7^. Furthermore, in a prospective proof-of-principle trial combining focal radiation therapy with granulocyte-macrophage colony-stimulating factor (GM-CSF) in metastatic patients with at least 3 measurable lesions, 11 of 41 patients (26.8%) had a systemic response^8^.

For over a decade, Demaria, Formenti and colleagues have been describing and clinically demonstrating the immune-priming potential of local radiotherapy and its ability to induce systemic anti-tumor immunity and regression of tumors outside the radiation field^9–13^. Radiation leads to immunogenic cell death, thereby allowing increased activation of key immune players including tumor antigen-specific effector cells^14,15^. Study of this phenomenon is now gaining traction among other groups^16,17^, yet why only certain patients experience abscopal responses remains unknown. Investigations into the mechanisms that govern systemic immunity are scarce, and an understanding of how we may be able to harness these mechanisms to guide targeted treatment for maximizing systemic reduction of total tumor burden remains elusive.

We present a conceptual study evaluating the systemic consequences of local perturbation of the tumor-immune ecosystem on each respective tumor site in a metastatic setting. We consider the global redistribution of activated T cells coupled with the local interactions of tumor and immune cells at each respective site, and attempt to gain insights into the role activated T cell dissemination may play in the broader context of systemic antitumor immunity. Metastatic environments featuring two or more anatomically distant tumor sites and the corresponding dynamic activation and distribution of immune effector cells are simulated, and a critical discussion of the study results and comparison to empirical clinical observations is provided. As high-resolution clinical data with which to evaluate all components of the highly complex interplay between host and immune system remain elusive, computational frameworks such as the one described herein may help to objectively assess the contribution of individual mechanisms to clinical outcomes.

Many of the parameters in the local tumor-immune interactions model have been previously described^18^, and are based on fitting the model to experimental data in mice^19^. This initial experiment involved the IV injection of BCL_1_ lymphoma tumor cells into BALB/c mice, and the longitudinal observation of the tumor cell number in the spleen. Mice were sacrificed and organs resected at various time points. Four groups of mice were included, with initial seeding populations ranging from 0.5 million to 50 million cancer cells. Before injection of cancer cells, mice in all groups received lethal whole body irradiation followed by bone marrow reconstitution using syngeneic or C57BL/6 bone marrow. Mean tumor cell count was obtained at sequential time points for each group over a period of approximately 120 days. Tumor dormancy was reached within 40 days in the group with syngeneic bone marrow reconstitution; in all other groups the tumor regressed following an initial growth period. The experimental data described and used for model fitting is shown in Supplementary Figure S1. Fitting of both the original Kuznetsov model and the model described in the present manuscript is also demonstrated in this figure.

A range of tumor cell population sizes will be considered in the present manuscript, with primary emphasis on those obtained from these murine experiments (which would likely correspond to small, subclinical disease in human patients). Clinically-detectable volumes on the scale of >1 cm will also be simulated. While it is conceivable that tumor-immune interactions are comparable between mouse and human, caution is warranted in assuming the applicability of results to the human clinical setting, and model calibration and validation on this scale must be rigorously performed as data becomes available.

## Methods

### Model development

The goal of the present study is to investigate the dynamic behavior of the interacting populations of tumor (*C*_*i*_) and immune (*E*_*i*_) cells in each respective site *i* of metastatic disease in both the presence and absence of therapy. Established models of both local tumor-immune dynamics^18^ and global T cell trafficking between metastatic sites^20^ are adapted and coupled to allow the simulation of multiple growing metastatic tumors.

Kuznetsov *et al*. described a pioneering model framework for describing interactions between cancer cells and cytotoxic T cells which was re-analyzed and modified for the present study^18^. Mechanisms with negligible biological and statistical contributions to the ability of the model to fit experimental data were omitted to reduce model complexity and the number of parameters (Supplementary Methods S1). We also note that the Kuznetsov model was originally developed and parameterized to simulate experimental tumor dormancy in which the host immune response is able to maintain the tumor at a constant volume^18^. For clinical relevance, tumor-immune dynamics need to be evaluated for growing tumors. Sensitivity analysis was conducted to identify the most influential parameters in the tumor-immune model, and the values of these parameters for which growth behavior is observed over the time period of interest were identified (Supplementary Methods S2). The adapted version of this experimentally parameterized model provides the foundation for the local tumor-immune interaction dynamics of the current study.

**Figure 1:**
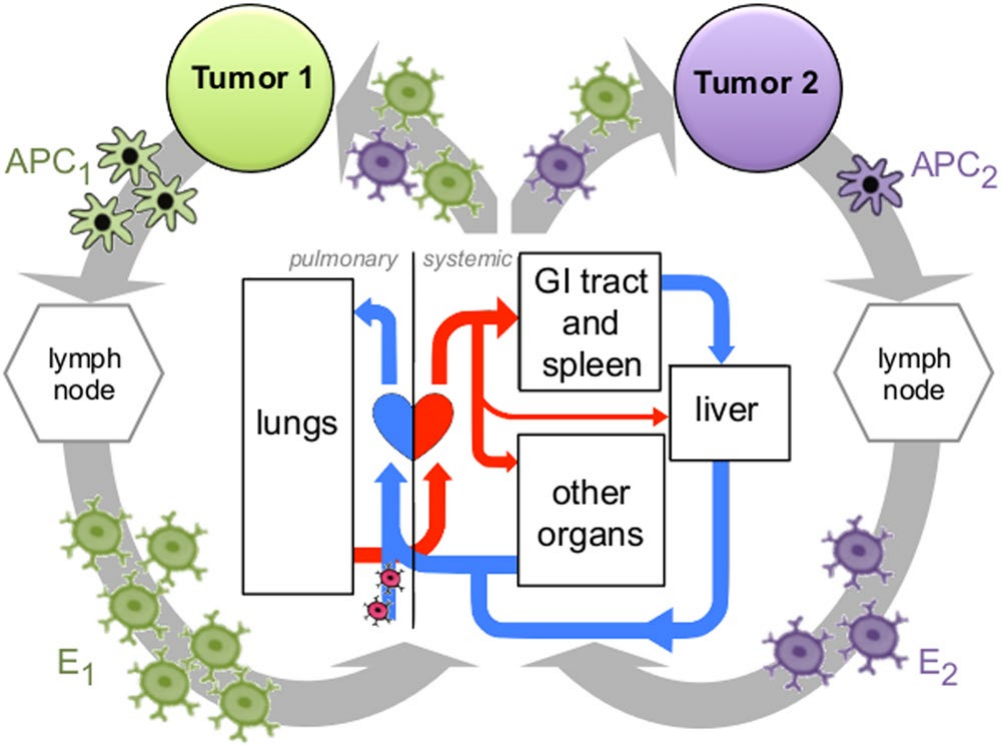
Schematic of the systemic interplay between metastatic tumors. At each respective tumor site *i*, antigen presenting cells (APC_i_) are being primed to activate a T cell mediated immune response. Following activation in the lymph node, effector T cells (E_i_) are trafficked through the circulatory system. Blood flows through the pulmonary circulation system before continuing systemically to distribute oxygen to cells throughout the body. This circulatory system allows T cells to pass through the host organs of any additional metastatic tumor sites; where overlap in antigenicity is present, effector cells activated at one tumor site in the patient may also extravasate at and surveil tumor sites elsewhere. The resulting distribution of immune cells depends on anatomical location of each metastasis, their respective volumes proportional to the tumor-bearing organs, and the blood flow fraction to each organ^19^.

Locally activated T cells have several potential targets in the metastatic setting (assuming overlap in antigenicity between a primary tumor and the metastases it seeds^21–23^, and can influence growth dynamics systemically. For a locally activated effector cell to extravasate at a particular anatomical target site it must first traverse the circulatory system to reach the tumor, influenced by both the blood flow to that region and activation site-specific homing cues^24^. Upon extravasation, T cells can then mount their cytotoxic attack on the local tumor cell population, in turn inducing cell death and continued immune activation and recruitment. A schematic of the systemic interactions of interest to the present study is provided in Fig. 1. The systemic distribution of locally activated cytotoxic T cells among *i* anatomically distant sites has been modeled analytically; a detailed description of this T cell trafficking model and related parameters have been previously described^20^. Site-specific assumptions including physiological blood flow fraction and average organ volume are detailed below.

Coupling adapted models of local dynamics and the systemic distribution of locally activated cytotoxic T cells among *i* anatomically distant metastatic sites leads to the following system of eqs {1, 2}:

1 $$\frac{d{C}_{i}}{dt}={\sigma }_{i}{C}_{i}(1-\frac{{C}_{i}}{{K}_{i}})-\lambda {C}_{i}{E}_{i}$$

2 $$\frac{d{E}_{i}}{dt}=\gamma ({E}^{\ast }-{E}_{i})+\sum _{j=1}^{N}{\omega }_{ij}(\vec{C})\frac{\delta {C}_{j}}{G+{C}_{j}}{E}_{j}$$

The population of cancer cells *C*_*i*_*(t)* at anatomical site *i* follows logistic growth with growth rate σ_*i*_ and organ-dependent carrying capacity *K*_*i*_, and is reduced by interaction with locally present immunocompetent effector cells *E*_*i*_*(t)* at rate *λ*. $\vec{C}$ denotes the vector $({C}_{1},\ldots ,{C}_{N})$ of cancer cell populations at each of N sites. The population of effector cells *E*_*i*_*(t)* at site *i* is recruited endogenously, with a target population E* of the observed physiological base number of effector cells obtained from the original model of Kuznetsov *et al*.^18^. For the conceptual study, were herein assume E* to be tissue independent. However, it is likely that E* would be tissue-specific as different organs have different risk levels for infection, for example, the skin and intestines are significantly more protected. T cells are recruited at rate *δ*, and *G* represents a half-maximal inhibitory parameter for new T cell recruitment. The probabilities of T cells activated at site *j* infiltrating site *i* are described by the matrix $\,{\omega }_{ij}$, as described previously^20^ and summarized in Supplementary Material S3. In brief, matrix $\,{\omega }_{ij}$ is a function of the product ${h}_{ij}\times \frac{BF{F}_{orga{n}_{i}}}{BF{F}_{compartmen{t}_{i}}}\times \frac{{V}_{i}}{{V}_{orga{n}_{i}}}$, where *BFF* notates the blood flow fraction to each respective tumor bearing organ and anatomic “compartment” (GI tract and spleen, liver, lungs, and other organs), *V* the volume of each tumor and organ, and *h*_*ij*_ the likelihood of extravasation at each respective metastasis (*i*) dependent on the site of activation (*j*)^20^. Lung, kidney and breast tumors were chosen due to their commonality, prevalence in metastatic disease and the availability of physiologic information including average organ size^25–28^ and oxygenated arterial blood flow fraction^20,29^, details of which are also provided in Supplementary Material S3. Homing parameters are imposed such that immune cells are 3× more likely to extravasate at their site of activation than elsewhere^20,24,30^.

The effect of surgery is modeled by an instantaneous removal of the entire tumor and immune populations in the treated site. To incorporate the effect of radiation therapy, *C*_*i*_*(t)* now denotes the viable tumor cell population before and after treatment, and we introduce ${R}_{i}(t)$ as the subpopulation of cancer cells that are lethally damaged by radiation and cleared from the tumor site at rate ε = 0.69 based on an approximate average apoptos is time of 24 hours^31^. Effects of acute radiation exposure are modeled by an instantaneous increase in ${R}_{i}$ equal to the decrease in *C*_*i*_ at each discrete treatment time point (9am, 5 days per week for 5 weeks, which is the conventional radiation fractionation scheme for most solid tumors), such that ${C}_{i}^{t+}=\,{C}_{i}^{t-}-\,\tau {C}_{i}^{t-}$ and ${R}_{i}^{t+}=\,{R}_{i}^{t-}+\,\tau {C}_{i}^{t-}$ at these instances. Here,$\,\tau$ is the proportion of cells lethally damaged by radiation and is calculated using the linear-quadratic (LQ) model with 1-exp($-\alpha d-\beta {d}^{2}$), where d (2 Gy) represents the radiation dose and *α* (Gy^−1^) and β (Gy^−2^) are tumor-specific radiosensitivity parameters with α/β = 10 Gy^32^. Instead of adding higher order extensions to the LQ model we solve the differential equations describing tumor-immune dynamics to account for biological events between radiation fractions^33^. The effect of varying these parameters is discussed in Supplementary Figure S5.

3 $$\frac{d{C}_{i}}{dt}={\sigma }_{i}{C}_{i}(1-\frac{{C}_{i}}{{K}_{i}})-\lambda {C}_{i}{E}_{i}$$

4 $$\frac{d{R}_{i}}{dt}=-\,\varepsilon {R}_{i}$$

5 $$\frac{d{E}_{i}}{dt}=\gamma ({E}^{\ast }-{E}_{i})+\sum _{j=1}^{N}{\omega }_{ij}(\vec{C},\vec{R})\frac{\delta {C}_{j}+{\delta }^{I}{R}_{j}}{G+({C}_{j}+{R}_{j})}{E}_{j}$$

The last term in eq. (5) now features an additional component representing the increased activation of T cells with rate ${\delta }^{I}$ = $2\delta \,\,$induced by radiation-induced cell death. This value is estimated based on the understanding that radiation-induced cell death can induce the release of tumor-derived antigens and danger signals that lead to increased attraction of APCs and priming of the immune system^10–13,34^. Note that the direct radiation-induced death of effector cells is not modeled explicitly; as we fit to experimental data, the radiation-induced immune recruitment is considered to represent the net effect of radiation on the immune system.

### Parameter estimation

From data fitting with the new, 6 parameter single-site model (Eqs (1, 2) for i = 1; Supplementary Methods S1) parameters comparable to those in the original Kuznetsov manuscript^18^ were obtained (Supplementary Table S1). We then identify values of the most sensitive parameters in the model for which monotonic tumor growth behavior is observed for the duration of our computational simulations (Supplementary Methods S2). The resulting parameters for the present study are then the tumor growth rate σ_*i*_ $\in (0.02,0.2)$, fixed tumor carrying capacity *K* = 5.14 × 10^8^, rate of cancer cell killing by T cells *λ* = 6.80 × 10^−8^, T cell decay or exhaustion rate *γ* = 0.25, T cell recruitment rate *δ* = 0.75, and a half-maximal inhibitory parameter for new T cell recruitment *G* = 5.7 × 10^4^. Rate parameters are given in units of 1/day unless otherwise specified. Note that in the present study the growth parameter selection technique prevents the scenario in which a tumor reaches maximum carrying capacity and growth completely saturates in the considered timespan. For this reason, while tumor growth rates σ may vary between different organs, the carrying capacity remains constant in all simulations. Parameter E^*^ = 0.3 × 10^6^ is the observed physiological base number of effector cells and is obtained from the original model of Kuznetsov *et al*.^18^, along with the aforementioned values of K, *λ* and *G*. Where simulations consider much larger tumor volumes than those found in these mouse experiments, adjustments to these parameters will be addressed in their respective sections.

### Model simulation

While metastatic seeding likely originates with only a small cluster of cells shed from the primary site, we are predominantly interested in the dynamic interactions that ensue when this secondary site is able to take hold and survive as a micrometastasis. Thus, for clear demonstration of the interplay between metastatic tumors and the effect of therapy, an initial tumor cell population of *C*_*i*_*(t* = *0)* = 5 × 10^5^ is imposed in all sites. An approximate T cell population in patients with tumors the size of those considered in the present study is not available from the literature. To approximate this value we initiate a simulation with 10 cancer cells (C(t = 0) = 10) and E(t = 0) = 0, and allow the populations of tumor and immune cells to co-evolve. Simulations were stopped when the tumor volume reached the values described experimentally, and the corresponding simulated immune cell population *E* = 4.3 × 10^5^ cells was used as initial condition for all simulations (Supplementary Figure S8). Numerical simulations are performed with ODE45 in MATLAB.

Anatomically distant sites with significantly different physiological blood flow fractions and thus T cell recruitment (lung and breast) are simulated both in isolation and upon metastatic seeding of the other after a period of 200 days. This allows qualitative and quantitative analysis of the influence of post-seeding T cell redistribution on both tumor and immune cell population dynamics at each of the two sites. Following this, the change in tumor volume of a primary site in the presence of each respective other site will be used to quantify the influence of systemic T cell redistribution and the new global immune environment on tumor growth. We will consider two cases: equal tumor growth rates and varying growth rates between tumor sites. Then we simulate cytotoxic and immunogenic consequences of surgery or radiation. All seeding and treatment simulations are repeated on the original Kuznetsov model calibrated for tumor dormancy for comparison.

## Results

### Metastatic seeding modulates primary tumor-immune dynamics through T cell re-routing

**Figure 2:**
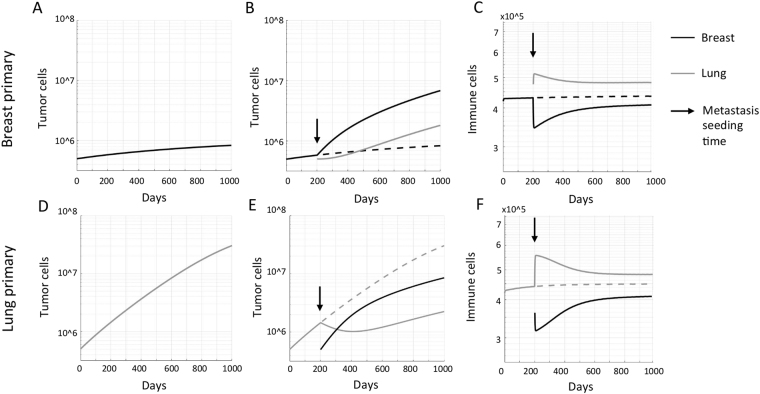
Consequences of metastatic seeding on primary tumor growth dynamics. (**A**) Simulated tumor growth of a breast tumor (with low growth rate σ = 0.03). **(B)** Seeding of a slightly faster-growing metastasis in the lung (σ = 0.035) at time t = 200 days yields accelerated growth of the primary breast tumor. **(C)** Metastatic seeding re-routes effector cells from the primary breast tumor to the lung metastasis due to its higher blood flow fraction. **(D)** Simulated tumor growth of a lung tumor (with growth rate σ = 0.035). **(E)** Seeding of a slightly slower-growing metastasis in the breast (σ = 0.03) at time t = 200 days yields transient decay followed by decelerated growth of the primary lung tumor. **(F)** Metastatic seeding increases the total systemic number of effector cells, and increases the number of immune cells extravasating at the primary lung tumor due to its higher blood flow fraction.

Simulated seeding of a faster-growing metastasis in the lung allows a slowly growing primary breast tumor to escape robust immune surveillance (Fig. 2A,B). This is attributable to a portion of the T cells activated in the breast now being redirected to the metastatic site; a significantly larger fraction of the circulating immune cells traffic through the pulmonary system, providing greater opportunity for extravasation at the lung (Fig. 2C). This redistribution induces a decrease in immune surveillance at the breast site and thus acceleration of tumor growth. If the lung tumor was assumed to be the primary, seeding of a slow growing metastasis in the breast would again increase the total number of effector immune cells in the circulation, with a larger number of cells extravasating in the lung (Fig. 2E). Thus, immune surveillance increases at the lung site in the presence of a breast tumor, inhibiting its otherwise rapid growth (Fig. 2D,E). Comparable simulations were conducted for the human scale with initial tumor volumes of *C*_*i*_*(t* = *0)* = 5 × 10^9^ and adapted carrying capacities of *K* = 5.14 × 10^11^, generating qualitatively comparable results (Supplementary Figure S9).

### Anatomically distant tumor growth rates determine change in primary tumor growth dynamics

**Figure 3:**
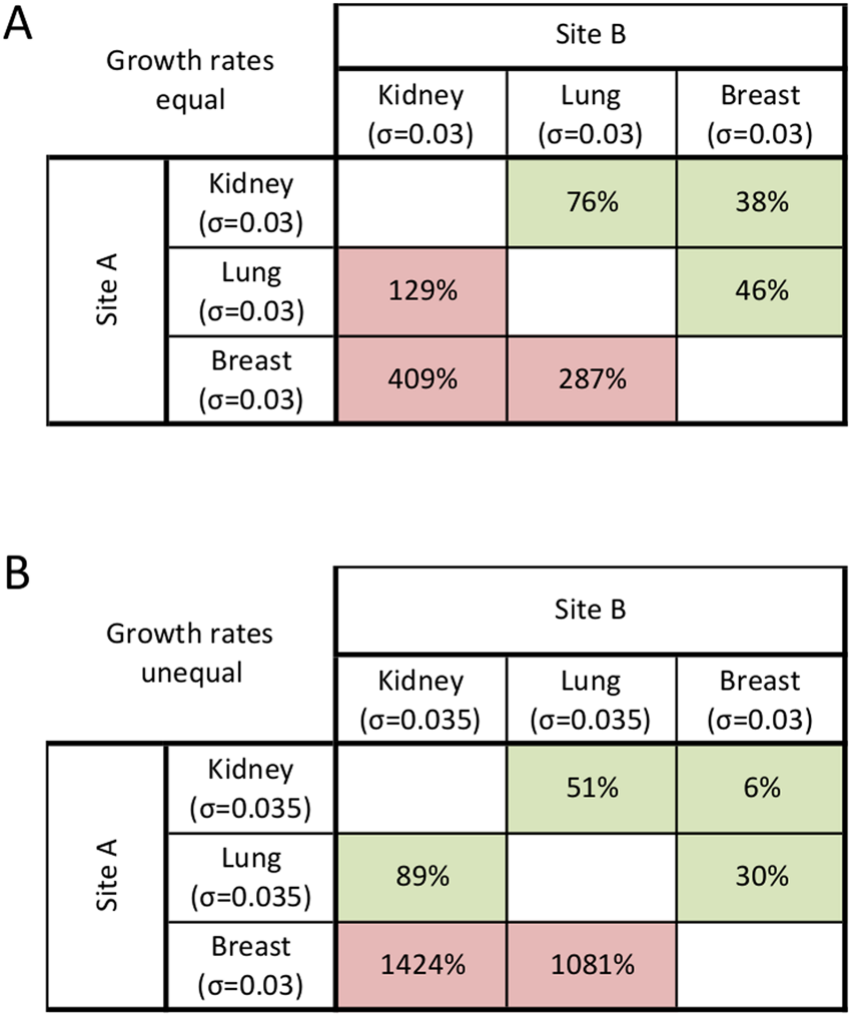
Changes in primary tumor volumes dependent on metastatic site recruitment index. Percentage change in volume of site A when a second tumor is seeded at site B. Sites are listed in order of decreasing recruitment index (RI). **(A)** Equal growth rates. If metastatic ‘Site B’ has a higher recruitment index than primary ‘Site A’ (*RI*_*B*_ > *RI*_*A*_), growth of the primary tumor will increase (red shade). If the metastatic site has a lower recruitment index than the primary site (*RI*_*B*_ < *RI*_*A*_), growth of the primary site will be decelerated (green shade). **(B)** Unequal growth rates. Even with only a small change in growth rate at one site, which tumors will experience growth or inhibition is no longer intuitively dependent on recruitment index or blood flow fraction.

Let us consider three tumor sites, kidney, lung and breast, with equal growth rates. We introduce a *recruitment index*, *RI*_*i*_, to quantify the model-predicted circulating T cell recruitment potential of each tumor site *i* dependent on blood flow fraction and tumor volume to organ volume ratio

6 $$R{I}_{i}=\,BF{F}_{organ}\times \frac{{V}_{C}}{{V}_{organ}}\times \frac{1}{z}$$

where *z* is a normalizing factor such that $\sum _{i}R{I}_{i}=1$ and *V*_*C*_ is an arbitrary tumor volume; then ∀ *V*_*C*_ < K RI_kidney_ > RI_lung_ > RI_breast_. Assuming equal growth rates are imposed in all metastatic sites (σ = 0.03), the primary tumor at site *i* will experience accelerated growth if the newly seeded tumor at an anatomically distant site *j* has a higher recruitment index *RI*_*j*_ > *RI*_*i*_. Conversely, if the newly seeded site has a lower recruitment index, growth of the primary tumor will be retarded (Fig. 3A). If tumors are seeded in all three considered organs, the largest tumor volume is observed for the tumor with the lowest recruitment index *RI*, and the order of tumor volumes is inversely proportional to the recruitment index, RI. This is a purely conceptual example; the systemic immune dynamics and the influence of metastatic seeding on a primary site are no longer intuitively derivable from blood flow fraction and recruitment index when tumor growth rates are unequal (Fig. 3B).

### Positive and negative abscopal effects after surgery

**Figure 4:**
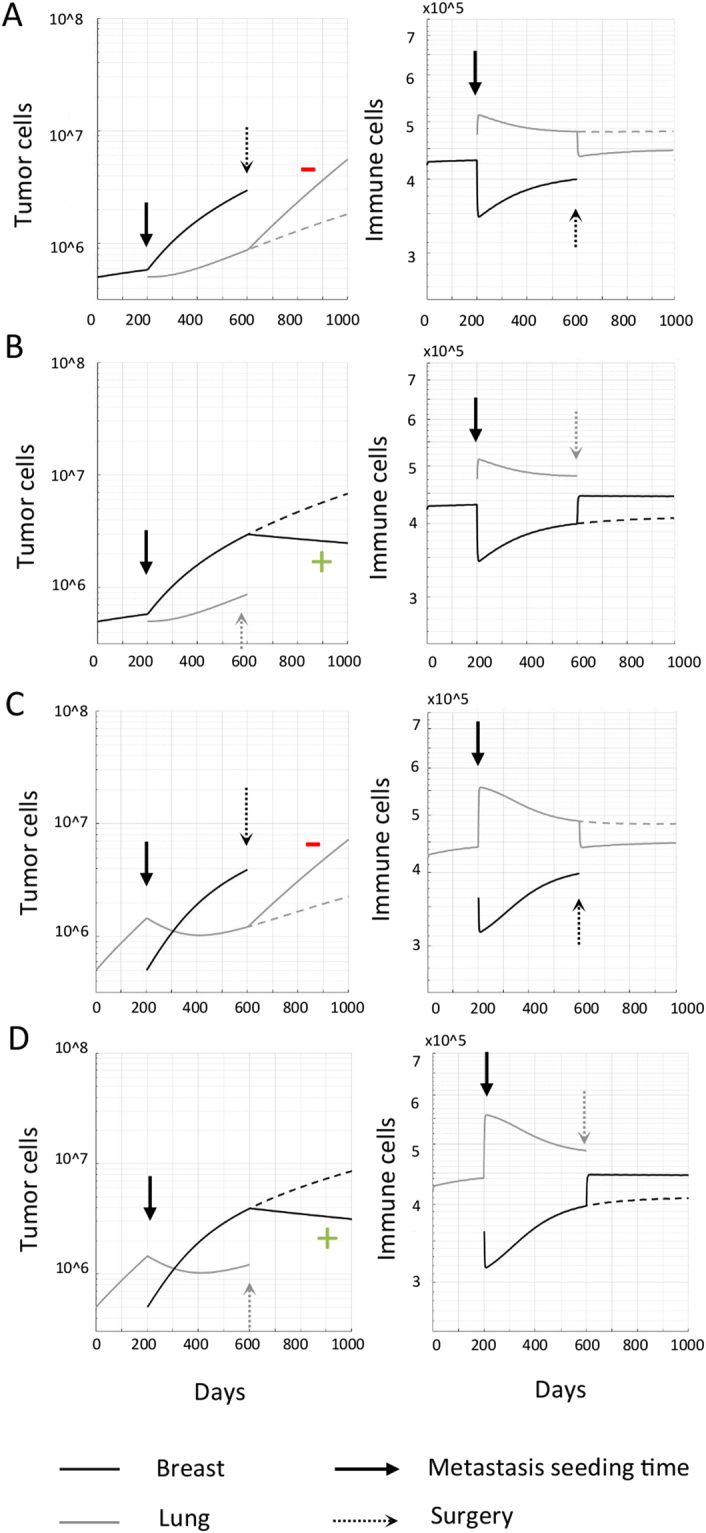
Abscopal effects of surgery. A primary tumor is seeded at time *t* = *0*. After 200 days, a second, metastatic site is seeded elsewhere. Following a period of simultaneous growth of both sites, the primary tumor is removed and abscopal responses scored in the distant tumor. **(A)** Primary breast tumor with metastasis in the lung. The lung tumor demonstrates rapid outgrowth (negative abscopal effect, red−) immediately following surgical removal of the breast tumor, which had previously been inhibiting the lung tumor by the redistribution of T cells activated by the breast to the lung. **(B)** Primary breast tumor with lung metastasis, and surgical removal of the lung tumor. The breast tumor is shrinking (abscopal effect, green+) immediately following surgical removal of the lung tumor, which had previously promoted breast tumor by the redistribution of T cells activated by the breast to the lung. **(C)** Primary lung tumor with breast metastasis, and surgical removal of the breast tumor. Systemic consequences as in panel A. **(D)** Primary lung tumor with metastasis in the breast. Systemic consequences as in panel B. Dashed lines show the trajectory of tumors without treatment.

Simulation results demonstrate that the abscopal effect of surgery depends on whether the growth of the untreated tumor(s) had previously been inhibited or promoted by the presence of the resected site. A tumor with a low recruitment index acts as a source of immune cells, generating more T cells to be redistributed systemically than it attracts, thereby inhibiting growth at anatomically distant sites. Thus, its removal may allow other tumors (with higher RI) to return to their intrinsic, faster growth trajectory, now without the additional systemic inhibition (Fig. 4A). Alternatively, tumors may experience a positive abscopal effect after surgical removal of a distant site with a higher RI that had previously served as a sink for trafficking immune cells (Fig. 4B). Model simulations suggest that tumor growth dynamics modulated by systemic immunity are independent of seeding order. Consequently, the described abscopal and negative abscopal responses occur irrespective of the surgically removed tumor being primary or metastasis (Fig. 4C,D).

### Abscopal effect after radiotherapy

**Figure 5:**
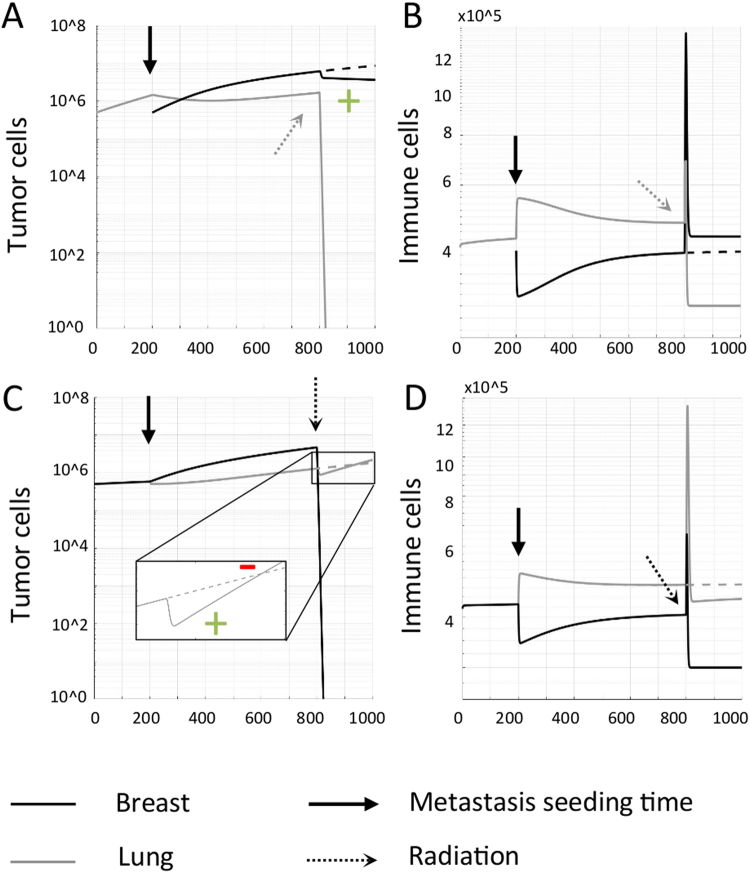
Abscopal responses after focal radiation. Abscopal responses in the untreated tumor are indicated by a green (+). Accelerated growth of an untreated metastatic tumor (negative abscopal response) is indicated by a red (−). A primary tumor grows and seeds a metastasis at time t = 200 days. Seeding order does not influence the respective tumor volumes at radiation time t = 800. **(A,B)** Radiation to the lung tumor with higher blood flow fraction. Irradiation of the lung tumor allows the previously redirected immune response to return to its site of activation in the breast, inducing a prolonged abscopal response here. **(C,D)** Radiation to the breast tumor with lower blood flow fraction. Irradiation of the breast tumor yields an initial, transient abscopal effect. At onset of radiation, the immune cell population initially increases at both sites due to radiation-induced immunity. As the breast tumor volume decreases, fewer immune cells are stimulated and the lung tumor is return to its intrinsic faster growth. This yields a transient decay followed by decelerated growth of the primary lung tumor (Fig. 2D).

Fractionated radiotherapy is simulated for a total dose of 50 Gy (5 days/week for 5 weeks, daily dose of 2 Gy at 9am). Unlike surgery, T cells are not instantaneously removed. Rather, immune cells are additionally induced with radiation (see recruitment term ${\delta }^{I}{R}_{j}$ in Eq. {5}). If the lung tumor with a higher blood flow fraction and recruitment index is irradiated, a higher proportion of immune cells will be available to surveil the breast site, inducing a volume decrease (positive abscopal effect; Fig. 5A,B). If the breast tumor with a lower blood flow fraction and recruitment index is irradiated, the initial release of radiation-induced immune cells yields a transient positive abscopal effect. With decreasing breast tumor volumes during radiation, the effect of radiation-induced antitumor immunity declines which enables accelerated lung tumor growth (negative abscopal effect; Fig. 5C,D).

## Discussion

The systemic effects of local treatment are becoming more widely documented in the clinical setting. A complete understanding of (i) which patients will response with systemic regression of metastases after local, often palliative treatment to an individual tumor site and (ii) how the mechanisms involved could be harnessed to improve treatment of metastatic cancer patients remains elusive. While arguments have been made that tumors are interconnected through long-range anti-angiogenesis signals^35^, recent advances suggest a role of the immune system^9^. Global tumor-immune dynamics may play a crucial role in understanding metastatic interdependence.

Here, we investigated the interconnectivity of anatomically distant tumors through systemic T cell redistribution among the tumor sites, based on clinical and experimental evidence that the abscopal effect after local radiation therapy is, at least in part, attributable to the dramatically increased priming and activation of effector cells. While several mathematical studies have been conducted into the mechanisms underlying metastatic seeding^36–38^, quantitative analysis of interconnectivity between existing sites is lacking. Building on earlier work that introduced the possibility of T cell redistribution influencing tumor-immune interactions systemically^20^, the present study is designed to quantify and compare these potential systemic effects in several clinically realistic scenarios. This involved extending earlier conceptual models of both local tumor-immune interactions and global T cell distribution to account for tumors in a phase of growth rather than dormancy, assessing how choice of treatment modality may influence systemic response, and evaluating simulation results in the context of actual clinical patient outcomes.

Concomitant immunity, the ability of a primary tumor to mount an immune response against a tumor and generate a memory that suppresses the growth of a secondary tumor seeded at a later time, has been demonstrated experimentally^39^, and subsequent discussions suggested that all tumors inhibit other tumors’ growth, with the extent of inhibition dependent on tumor volume^40^. These results suggests that one site may either inhibit *or promote* a second, depending on the patient-specific anatomical distribution of metastatic disease. Seeding new metastatic sites could either facilitate or restrict growth of existing tumors, depending on whether the newly seeded site recruits T cells that were originally surveilling elsewhere or produces T cells that are more likely to extravasate elsewhere in the body. This implies that tumors could be held in a dormant state by the presence of distant sites capable of producing a substantial immune response that propagates systemically.

Based on this understanding, if an inhibiting site were to be instantaneously removed, for example during surgical resection, a subclinical cancer cell deposit, micrometastasis or immune-modulated dormant tumor may no longer be constrained. Rapid outgrowth of anatomically distant tumors following surgical resection of a tumor has been observed clinically^41^. While new metastatic sites could be seeded after the removal of a primary tumor, it is conceivable (and mathematically more likely^9^) that these sites were already present but sub-clinically dormant, and that removal of systemic immune surveillance initiated by the primary permitted their rapid escape from dormancy. In addition to positive abscopal effects (metastatic decline), the described model can also reproduce such negative (metastatic outgrowth) systemic effects depending on the patient-specific metastatic setting.

For clinical applicability we adapted the mathematical model calibrated for tumor dormancy to simulate tumor growth. The suggested systemic implications of local therapy, however, are also observable in the original model framework (Supplementary Figure 6). Based on the principle of newly seeded metastases having the ability to not only activate additional effector cells but also redirect an existing immune response away from a primary tumor, the potential arises for continuous seeding of (potentially subclinical) metastatic sites to allow escape from dormancy at a previously controlled site (Supplementary Figure 7), assuming a physiological maximum activation capacity of effector cells has not been reached. This potential for metastatic seeding to escape from dormancy could cast new light onto our current understanding of the mechanisms governing tumor growth.

Although surgery can significantly reduce tumor burden, it is not curative in the metastatic setting. Surgery additionally causes the instantaneous removal of a potentially large number of immune cells currently active at the local site, and could induce rapid outgrowth of a metastatic site. Radiation, in contrast, could provide increased activation of tumor-specific T cells that have the potential to attack distant metastatic tumors and vaccinate against future disease, in addition to local tumor eradication^42^. In a recent retrospective analysis of radiotherapy sequencing for early stage breast cancer patients, neoadjuvant radiation prior to surgery has been shown to significantly reduce the risk of second cancer diagnoses by 12% over 20-years follow up^43^. This is in line with studies of intratumoral enrichment of immune cells in surgically resected samples of rectal patients who received neoadjuvant radiotherapy^44^. Local radiation may convert the tumor into an *in situ* vaccine that facilitates abscopal regression of clinically overt or subclinical tumors^10,12^.

The present manuscript focuses on tumors initiated with as few as 5 × 10^5^ cells; qualitatively similar results are observed in larger tumors of the order of 10^11^ cells. A thorough analysis of the dynamics of multiple metastases of all sizes was beyond the scope of this manuscript, yet intuitively the same principles could apply across spatial scales when properly calibrated for human tumor-immune interaction dynamics. The described global redistribution of immune effector cells may either contribute to the eradication of metastases prior to reaching a clinically detectable volume, as is assumed to be the case in the vast majority of seeding occurrences, or promote their rapid growth. Even if tumor volumes are clinically detectable, for patients with widely metastatic disease such abscopal effects may be too subtle to be detectable in all tumor sites. While we highlight herein cases in which clinically observable changes in metastatic tumor volume result from local treatment, simulations with different parameter combinations may yield systemic changes that are too small to be detected clinically. Only in specific conditions, and dependent on the site receiving treatment, do model predictions suggest the magnitude of these effects will be measurable to the clinician. This may, at least in part, offer justification why clinically measurable abscopal responses remain rare. Our results also suggest that abscopal effects could indeed be transient and only be detected if clinical monitoring has sufficiently high temporal resolution. Additionally, removal of a single tumor site and perturbation of the global immune population may shift remaining tumors back onto their individual growth trajectories. Assessment of response by RECIST (Response Evaluation Criteria in Solid Tumors) could completely miss such abscopal effects. Although tumors may progress and continue to grow, a change in their rate of growth constitutes an abscopal response. Thus, the number of systemic responses after focal therapy occurring in the clinical setting may be significantly higher then previously thought.

There are undoubtedly many contributing mechanisms to systemic antitumor immunity; several simplifying assumptions were made in this conceptual study. Cytotoxic T cells are just one component of the host immune response. Based on various experimental and clinical studies demonstrating the ability of radiotherapy to increase priming and proliferation of cytotoxic T cells, inducing greater systemic circulation and increased potential for eradicating metastasis^12,14,15^, understanding the role activated T cell dissemination may play in the broader context of systemic immune responses can provide motivation for further studies both in the laboratory and in the clinic before further complicating assumptions are incorporated. Variation in antigenicity between metastatic sites could be more accurately quantified if relevant patient-specific information were available, and the efficacy of antigen presenting cells is likely to influence clinical outcomes. Several parameters relating to the interaction of tumor and immune cells described herein were obtained from fitting to the data of experimental studies in mice in the absence of equivalent data for human patients. Parameter re-evaluation could be conducted in the future for validation; while exact tumor volume predictions may then vary, the fundamental concepts of growth promotion and inhibition induced by systemic immune interconnectivity between sites are independent of parameter choice, and qualitatively similar results would be expected. Importantly, a further extension to the current model would be to incorporate the local expansion of the activated effector cell population at each respective tumor site. Microenvironmental inhibition or promotion of immune proliferation and expansion at a particular site could strongly influence the outcome of these systemic tumor-immune dynamics. Without clinical information with which to parameterize such a term, this mechanism has been omitted from the present work.

The purpose of the present work is to provide a theoretical demonstration of the previously unappreciated systemic consequences of local therapy. Despite the inherent simplifications of the described model, based on our understanding of the systemic nature of host immune response, the potential for perturbation of the local tumor-immune ecosystem to have significant implications for tumor-immune interactions elsewhere in the body is clear. If metastatic sites are interconnected through the immune system, truly local therapy does not exist. Assuming not all sites participate in systemic immune surveillance equally, while treating one metastatic site could yield a positive abscopal response, treatment at other sites could trigger aggressive outgrowth of an untreated tumor. Further study is certainly warranted to conclusively identify individual mechanisms influencing the direction and magnitude of these non-random abscopal responses in cancer patients.

## Electronic supplementary material

Supplementary Material
