# Supplementary material for: Immune interconnectivity of anatomically distant tumors as a potential mediator of systemic responses to local therapy
Source: Sci Rep. 2018 Jun 21;8:9474. doi: 10.1038/s41598-018-27718-1 (PMC6013469; doi:10.1038/s41598-018-27718-1)
Supplement: Supplementary file 1 — Supplementary Material [file 41598_2018_27718_MOESM1_ESM.pdf]

Immune interconnectivity of anatomically distant tumors as a potential mediator of systemic responses to local therapy

Rachel Walker[1], Jan Poleszczuk[2], Shari Pilon-Thomas[3], Sungjune Kim[4], Alexander A.R.A. Anderson[1], Brian J. Czerniecki[5], Louis B. Harrison[4], Eduardo G. Moros[4], Heiko Enderling[1,4,*]

Departments of [1]Integrated Mathematical Oncology, [3]Immunology, [4]Radiation Oncology, [5]Breast Oncology, H. Lee Moffitt Cancer Center and Research Institute, Tampa, FL, USA
[2]Nalecz Institute of Biocybernetics and Biomedical Engineering, Polish Academy of Sciences, Warsaw, Poland

[*]Correspondence: Heiko.Enderling@moffitt.org

Supplementary Material

S1. Reduction of model complexity

The original model framework of Kuznetsov et al. introduced a term describing the rate (*1-p*) at which immune cells die in interactions with tumor cells, the optimal value of which was found to be 0.002.[1] Repeating the model fitting to experimental data presented in the original manuscript[1,2] in the presence and absence of this term (i.e. with (*1-p)* equal to 0.002 and 0, respectively), demonstrated an almost identical ability of the model to qualitatively represent the data when this term is removed (**Figures 1, 2**). To quantify the benefit of omitting this term the Akaike information criterion[3] (AIC) could be used to measure the relative "quality" of each version of the model (a = original 7 parameter model, b = new 6 parameter model). The AIC evaluates the trade-off between model complexity in terms of number of parameters, and the accuracy of model fit for a specified data set, and can be calculated as follows:

$$AIC = n * \ln\left(\frac{RSS}{n}\right) + 2 * M + (2 * M * (M + 1))/(n - M - 1) \tag{7}$$

where n is the number of data points (observations), M is the number of parameters in the model, and the latter term provides a bias-adjustment for small sample sizes. The first term represents the log likelihood of each model, approximated by the residual sum of squares. AIC analysis suggests that the simpler model with one less parameter is the "better" model with a relative likelihood of Ω_b = 0.9977 versus Ω_a = 0.0023, where $\Omega_i = \exp(-0.5\,\Delta_i)/\sum_i^R \exp(-0.5\,\Delta_i)$, R the number of models being compared, and $\Delta_i = AIC_i - \min(AIC)$.[3] This suggests that the 6 parameter model (model b) is comparably representative of the data while reducing risk of overfitting. Parameters obtained from fitting the original data set to this new model are provided in **Table S1**, along with the comparable values from the original model of Kuznetsov et al. Based on this comparison, the simpler model was used for the purposes of this manuscript.

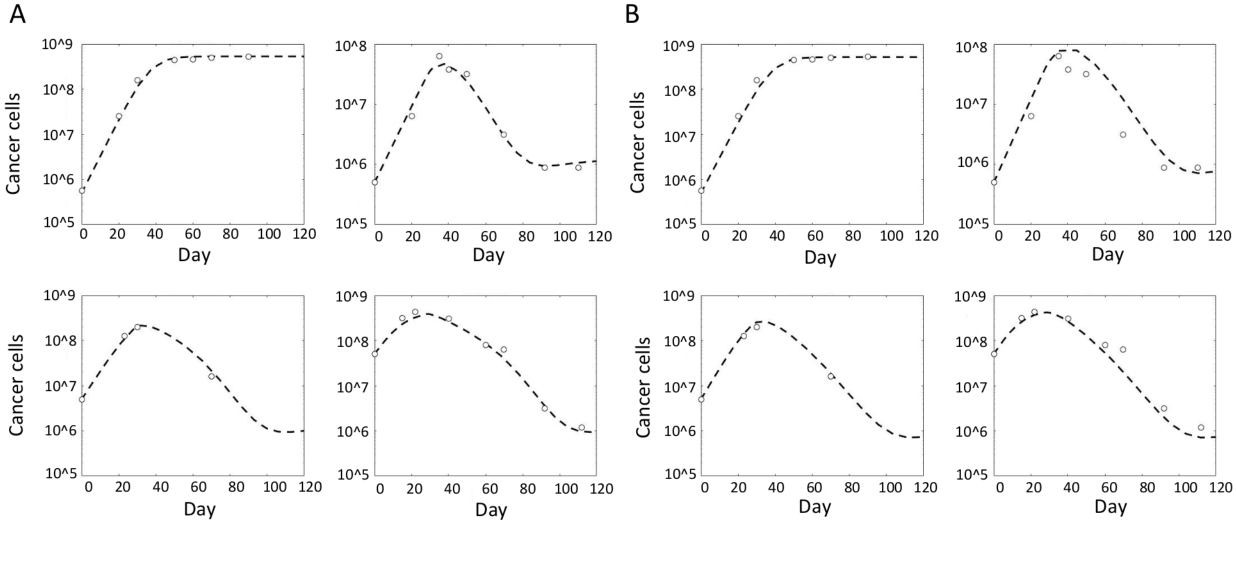

Figure S1. Model fitting to approximate values of experimental data of Siu et al.[2] as presented in Kuznetsov et al.[1] for models a (7 parameter, panel A) and b (6 parameter, panel B). Empty circles represent data points, dashed line is model fit.

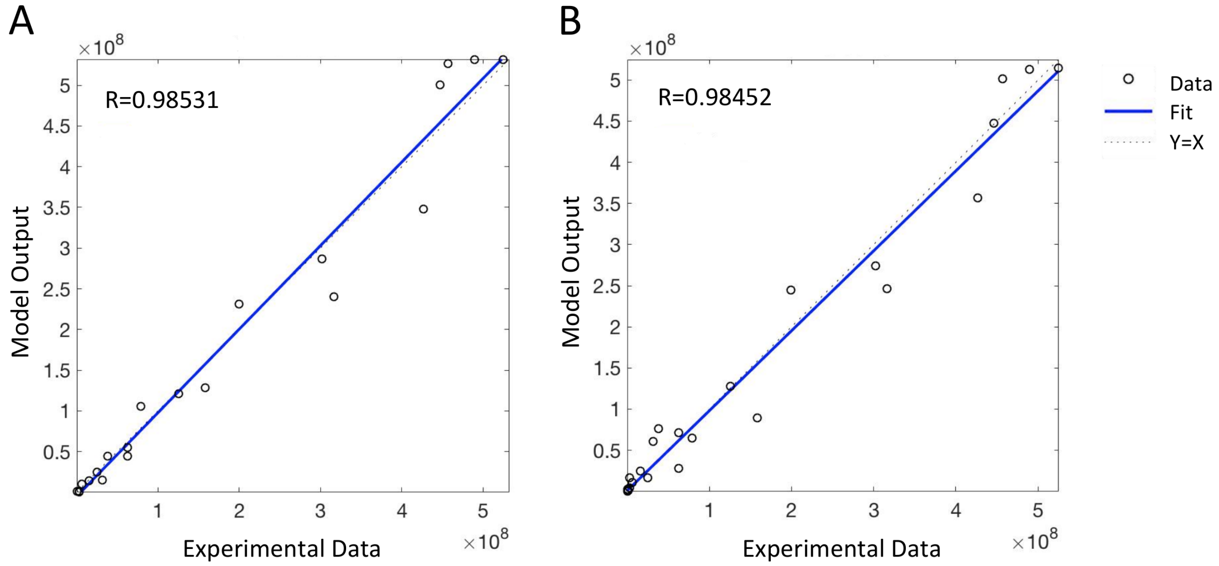

Figure S2. R^2 values for model fitting to approximate values of experimental data of Siu et al.[2] as presented in Kuznetsov et al.[1] for models a (7 parameter, panel A) and b (6 parameter, panel B). Regression line is shown in blue.

Model	σ	K	λ	γ	δ	G	p
a	0.188	5.32E+08	1.39E-07	0.59	0.53	1.60E+05	9.98E-01
b	0.181	5.14E+08	6.80E-08	0.74	0.69	5.70E+04	N/A

Table S1. Parameters identified from data fitting with the new, 6 parameter model (b) as compared to the original Kuznetsov model (a).[1]

S2. Identification of growth parameters

Sensitivity analysis was conducted to identify the parameters for which a small perturbation induces the largest change in the dynamics of the system.[4] Relative local sensitivities S_i were calculated for each parameter *i* using the standard definition

$$S_i = \frac{\partial \log C}{\partial \, log p_i} = \frac{dC/C}{dp_i/p_i} \quad (8)$$

where *C* is the cancer cell population and p_i the *i*th parameter.[5] Approximations of the derivative were calculated using the second-order central finite differencing method. These logarithmic sensitivities were calculated locally (based on perturbations to each parameter of ±1%) for the default parameter values and for 10,000 random parameter sets generated using Latin hypercube sampling.[4,6] All sensitivities were calculated at 10 evenly spaced time points during a 1000 day simulation period and averaged prior to ranking. Parameters δ, γ, and σ and were found to lie within the top 3 most sensitive parameters in 92%, 90% and 58% of the 10,000

simulations, respectively (**Figure S3**), with δ most commonly ranking 1st, γ most commonly ranking 2nd, and α most commonly ranking 3rd.

A parameter sweep was then conducted to identify the values of these parameters that could induce constant growth behavior over our time period of interest (1000 days). This involved allowing each parameter to vary from 0 to 1 in increments of 0.05. Simulations were run with all combinations of the three parameters (N=200^3), and combinations for which $C(t+1) > C(t)\ \forall\ t \in (0{,}1000)$ were identified. These parameter combinations and the distribution of each parameter within these combinations are shown in **Figure S4**. We arbitrarily select δ = 0.25, γ = 0.75 and $\sigma \in (0.02{,}0.2)$ for presentation of results in the present manuscript, although other combinations within these distributions could have been equally demonstrative.

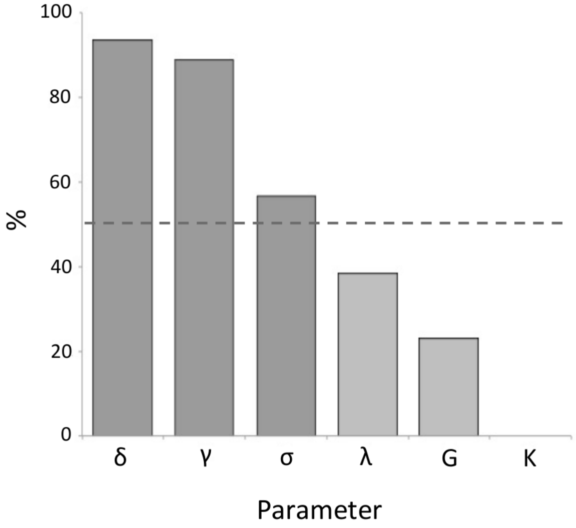

Figure S3. Proportion of all simulations for which each respective parameter ranked 1st, 2nd, or 3rd.

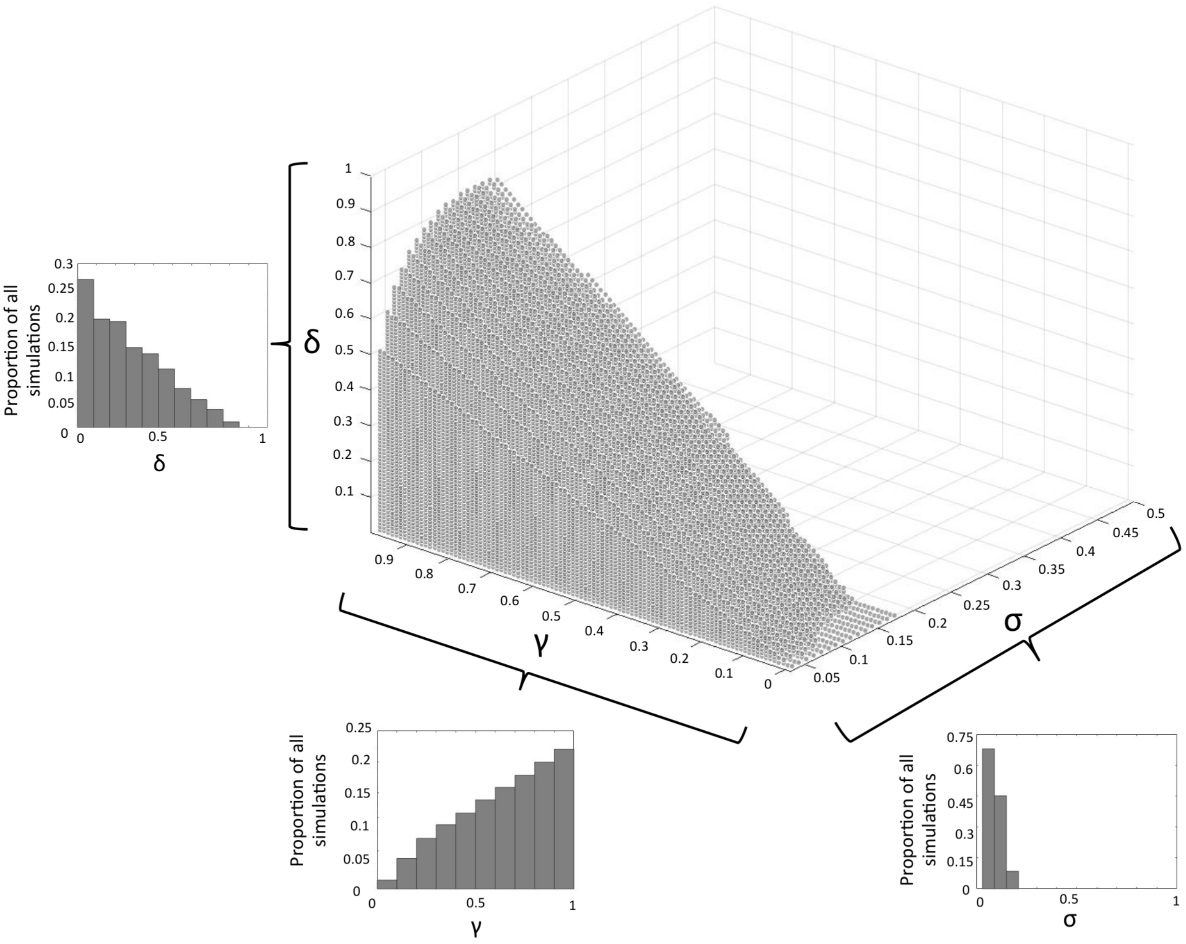

Figure S4. Distribution of all combinations of parameters (α, γ, δ) for which continuous growth behavior was observed over our time period of interest.

S3. *Recruitment term*

The following description is taken from Poleszczuk et al. and the reader is directed to [7] for additional detail. Four compartments (COMPs) of the blood system are described: pulmonary circulation (LU); gastro-intestinal tract and spleen (GIS); liver (LI); and all other organs in the systemic circulation such as breast or kidney (SO). Matrix ω_{ij} describes the probabilities of T cells activated at site *j* infiltrating site *i*, and is given by

$$\omega_{ij} = \frac{W_{COMP_i} P_{ij}}{H_{COMP_i}} \tag{9}$$

where $W_{COMP} = A/\Delta$ with

$$A = H_{LU} \quad \text{if} \quad COMP = LU \tag{10}$$

$$A = H_{LI}(1 - H_{LU})(BFF_{LI} + BFF_{GIS}(1 - H_{GIS})) \quad \text{if} \quad COMP = LI \tag{11}$$

$$A = H_{GIS} BFF_{GIS}(1 - H_{LU}) \quad \text{if} \quad COMP = GIS \tag{12}$$

$$A = H_{SO} BFF_{SO}(1 - H_{LU}) \qquad \text{if} \qquad COMP = SO \tag{13}$$

and Δ is a normalizing constant such that the sum of absorption probabilities over all four compartments is equal to one. BFF_{COMP} is the blood flow fraction to the respective compartment, and V_i is the volume of the ith organ. Probability P_{ij} is the probability that a T cell activated at site *j* will infiltrate the ith tumor site after entering a given compartment. This is calculated by the probability that a T cell will flow through a tumor site (approximated by relative blood flow through the tumor bearing organ multiplied by the fraction of the organ populated b the tumor), multiplied by h_{ij}, the probability of extravasation from the blood into the tissue:

$$P_{ij} = h_{ij} \times \frac{BFF_{organ_i}}{BFF_{compartment_i}} \times \frac{V_i}{V_{organ_i}} \tag{14}$$

Here, $h_{ij} = h_a$ if the T cell was activated in the given organ ($organ_i = organ_j$) and $h_{ij} = h_n$ otherwise ($1 \geq h_a \geq h_n$). Finally, H_{COMP} is the probability that a T cell will extravasate at one of the metastatic sites *after entering a given compartment:*

$$H_{COMP} = \sum_{i=1}^{N_{COMP}} P_{ij} \tag{15}$$

Relevant parameters for our tumor sites of interest are as follows: (V_{lung} = 3679 mL, V_{kidney} = 249 mL, V_{breast} = 500 mL)[8-11] and oxygenated arterial blood flow fraction (BFF_{lung} = 100%, BFF_{kidney} = 8.5%, BFF_{breast} = 2%)[7,12].

S4. Radiation-associated parameters

Based on observations in the breast, α = 0.3 and α/β = 10 were arbitrarily chosen for demonstrative purposes.[13] **Figure S5** demonstrates the influence of varying these radiation-associated parameters on model output. While the model appears to be relatively insensitive to the value of the ratio α/β, the specific α value can have a more significant effect. For future testing, site-specific LQ parameters should be determined for each respective metastatic site of interest for maximum accuracy.

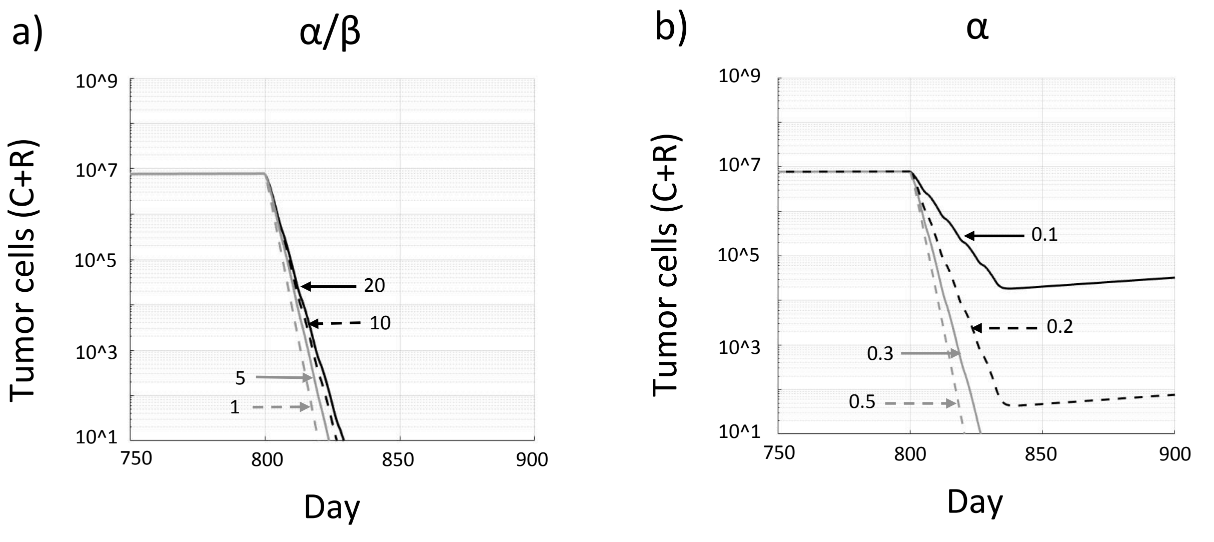

Figure S5. Influence of variation of each respective radiation-associated parameter on model outcomes.

S5. Dormancy-associated results

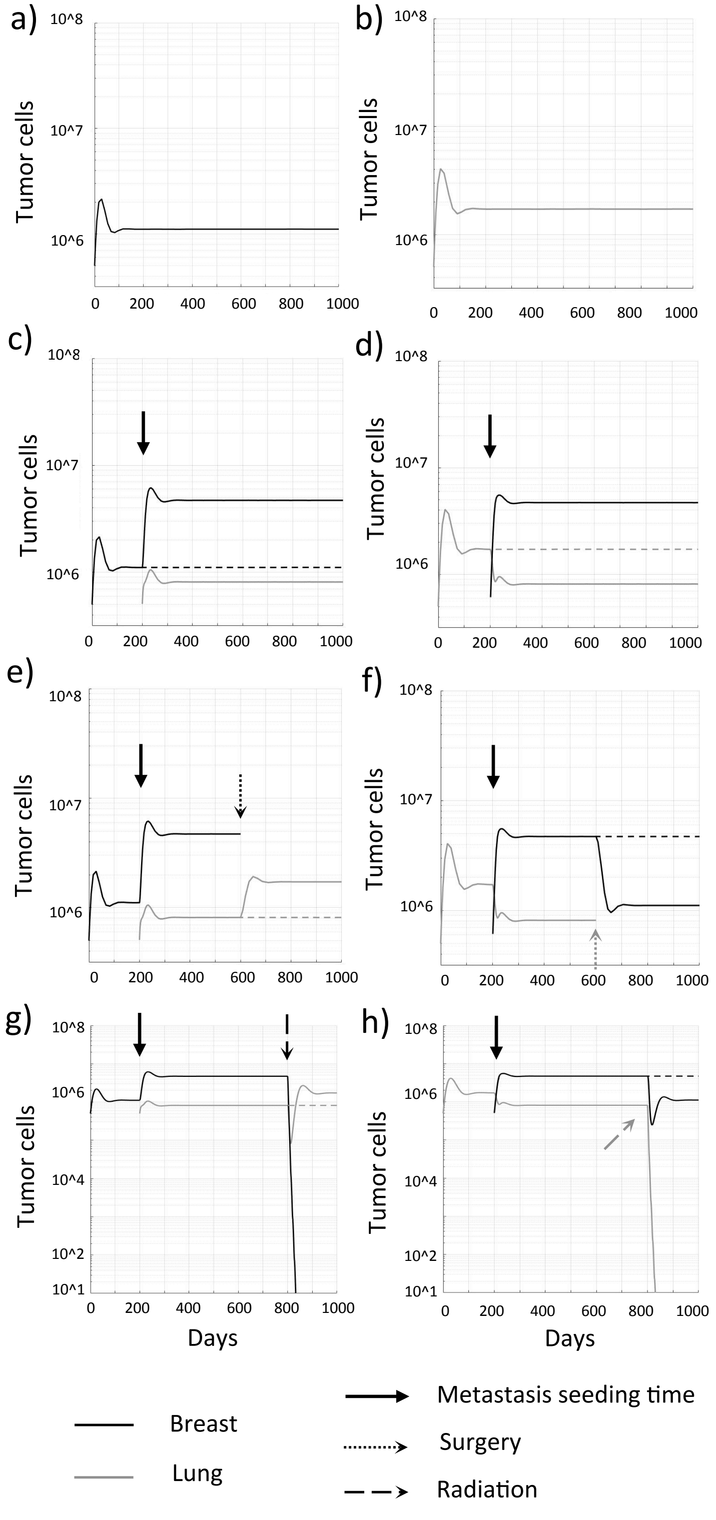

Figure S6. Overview of results in the setting of tumor dormancy. Figures **a,b)** demonstrate the initial transient growth and subsequent host immune response at seeding of individual primary tumors (breast and lung, respectively), followed by a dormant state in which the tumor and host immune system are in equilibrium. Upon seeding of a second site (lung and breast, respectively)

an increase in the dormant volume of the breast tumor is observed along with a corresponding decrease in the dormant volume of the lung tumor attributable to the redistribution of activated effector cells to the site of highest recruitment index, as demonstrated in the growth setting **(c,d).** Resection of a breast primary results in an increased dormant volume of a lung metastasis **(e),** attributable to a decrease in immune surveillance at the lung site. Resection of the lung metastasis results in significantly decreased dormant volume of a breast primary **(f),** attributable to an increase in immune surveillance at the breast site**.** Irradiation of the breast site **(g)** results in not only shrinkage of this site, but also an initial, transient abscopal effect prior to the lung established an equilibrume volume greater than that in the presence of the breast tumor. Irradiation of the lung site **(h)** allows the previously redirected immune response to return to its site of activation in the breast, inducing a prolonged abscopal response here. In all dormancy modeling, parameters were as defined in Table 1 for the 6 parameter model.

S6. Escape from dormancy

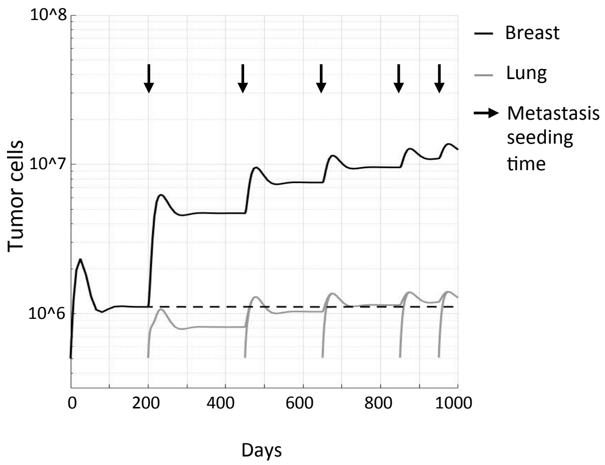

Figure S7. In the case of a primary breast tumor having reached a dormant equilibrium with the host immune system, the seeding of an additional site in the lung induces both an increase in the number of activated effector cells circulating systemically, and the redistribution of effector cells previously surveilling the breast site to the new lung metastasis. This has the potential to reduce the immune surveillance at the breast site, inducing a transient period of growth until dormancy is re-established at a higher equilibrium volume. This pattern can be repeated until such point that the immune surveillance of the breast site reaches a minimum, or the physiologic base level of T cell surveillance in healthy tissue. This demonstrates the feasibility of an metastasis-seeding induced escape from dormancy mechanism, distinct from previously described processes of tumor growth.

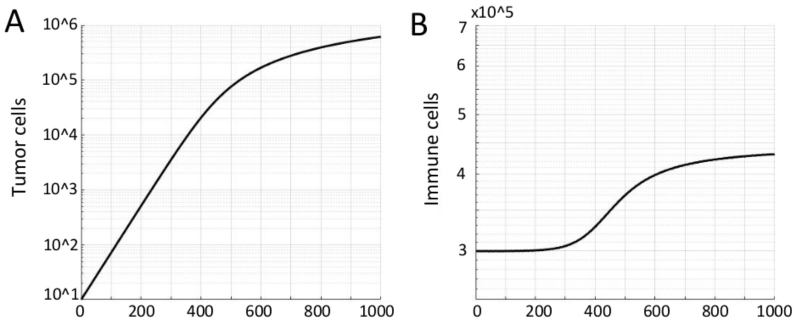

Figure S8 A. Simulations are initialized with a tumor cell population of C_i = 10 and run until a population of $5x10^5$ cells is reached. **B**. At this time, the effector cell population is $E_i = 4.3x10^5$. This motivates the choice of $E_i\,(t{=}0) = 4.3x10^5$ used as an initial condition in the simulations described in this manuscript.

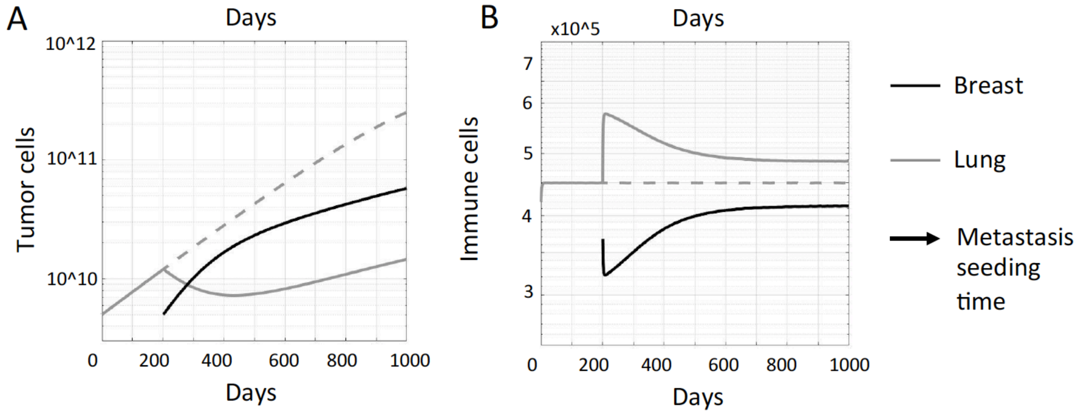

Figure S9. A. Seeding of a slower-growing metastasis in the breast (σ = 0.03) after initiation of a faster-growing primary in the lung (σ = 0.035) yields transient decay followed by decelerated growth of the primary lung tumor. **B.** Metastatic seeding increases the total systemic number of effector cells, and increases the number of immune cells extravasating at the primary lung tumor due to its higher blood flow fraction. These results with initial tumor volume of $C_i(t{=}0){=}5x10^9$ are qualitatively similar to those observed with an initial tumor volume of $C_i(t{=}0){=}5x10^5$ (**Figure 2**), suggesting the results presented herein do not depend on choice of initial tumor volume.

References

1. Kuznetsov, V.A., Knott, G.C. Modeling tumor regrowth and immunotherapy. *Math and Computer Modelling.* **33**, 1275-1287 (2001)

2. Siu, H., et al. Tumor dormancy I: Regression of BCL_1 and induction of a dormant tumor state in mice chimeric at the major histocompatibility complex. *J Immunol.* **137**, 1376-1382 (1986)

3. Hu, S. Akaike information criterion. Center for Research in Scientific Computation. North Carolina State University. http://www4.ncsu.edu/~shu3/Presentation/AIC.pdf (2007)

4. Nagaraja, S., et al. Computational approach to characterize causative factors and molecular indicators of chronic wound inflammation. *J Immunol.* **192(4)**, 1824-34 (2014)

5. Hamby, D.M. A review of techniques for parameter sensitivity analysis of environmental models. *Environ Monit Assess.* **32.2**, 135-154. (1994)

6. Iman, R.L. Latin Hypercube Sampling in *Encyclopedia of Quantitative Risk Analysis and Assessment III* (ed. Melnick, E.L.) (Wiley, 2008).

7. Poleszczuk, J., et al. Abscopal benefits of localized radiotherapy depend on activated T cell trafficking and distribution between metastatic lesions. *Cancer Res.* **76**, 1009 (2016)

8. Abduljalil, K., et al. Anatomical, physiological and metabolic changes with gestational age during normal pregnancy: a database for parameters required in physiologically based pharmacokinetic modelling. *Clin pharmacokinet*. **51(6)**, 365-96 (2012)

9. Henderson, J.M., et al. Measurement of liver and spleen volume by computed tomography. Assessment of reproducibility and changes found following a selective distal splenorenal shunt. *Radiology.* **141(2)**, 525-7 (1981)

10. Puybasset, L., et al. A computed tomography scan assessment of regional lung volume in acute lung injury. The CT Scan ARDS Study Group. *Am J Respir Crit Care Med.* **158(5 Pt 1)**, 1644-55 (1998)

11. Poggio, E.D., et al. Donor kidney volume and outcomes following live donor kidney transplantation. *Am J Transplant*. **6(3)**, 616-24. (2006)

12. Valentin J. Basic anatomical and physiological data for use in radiological protection: reference values. *Ann ICRP.* **32(3–4)**, 1-277. (2002)

13. Williams, M.V., Denekamp, J., Fowler, J.F. A review of alpha/beta ratios for experimental tumors: implications for clinical studies of altered fractionation. *Int J Radiat Oncol Biol Phys.* **11(1)**, 87-96 (1985)
